# Correlation of the National Emergency Medicine M4 Clerkship Examination with USMLE Examination Performance

**DOI:** 10.5811/westjem.2015.10.25496

**Published:** 2015-12-14

**Authors:** Luan E. Lawson, Davis Musick, Kori Brewer

**Affiliations:** * East Carolina University, Brody School of Medicine, Department of Emergency Medicine, Greenville, North Carolina; † Virginia Tech Carillion School of Medicine, Department of Internal Medicine, Roanoke, Virginia; ‡ East Carolina University, Brody School of Medicine, Department of Medical Education, Greenville, North Carolina

## Abstract

**Introduction:**

Assessment of medical students’ knowledge in clinical settings is complex yet essential to the learning process. Clinical clerkships use various types of written examinations to objectively test medical knowledge within a given discipline. Within emergency medicine (EM), a new national standardized exam was developed to test medical knowledge in this specialty. Evaluation of the psychometric properties of a new examination is an important issue to address during test development and use. Studies have shown that student performance on selected standardized exams will reveal students’ strengths and/or weaknesses, so that effective remedial efforts can be implemented. Our study sought to address these issues by examining the association of scores on the new EM national exam with other standardized exam scores.

**Methods:**

From August 2011 to April 2013, average National EM M4 examination scores of fourth-year medical students taken at the end of a required EM clerkship were compiled. We examined the correlation of the National EM M4 examination with the scores of initial attempts of the United States Medical Licensing Exam (USMLE) Step 1 and Step 2 Clinical Knowledge (CK) examinations. Correlation coefficients and 95% confidence intervals of correlation coefficients are reported. We also examined the association between the national EM M4 examination score, final grades for the EM rotation, and USMLE Step 1 and Step 2 CK scores.

**Results:**

133 students were included in the study and achieved a mean score of 79.5 SD 8.0 on the National EM M4 exam compared to a national mean of 79.7 SD 3.89. The mean USMLE Step 1 score was 226.8 SD 19.3. The mean USMLE Step 2 CK score was 238.5 SD 18.9. National EM M4 examination scores showed moderate correlation with both USMLE Step 1 (mean score=226.8; correlation coefficient=0.50; 95% CI [0.28–0.67]) and USMLE Step 2 CK (mean score=238.5; correlation coefficient=0.47; 95% CI [0.25–0.65]). Students scoring below the median on the national EM M4 exam also scored well below their colleagues on USMLE exams.

**Conclusion:**

The moderate correlation of the national EM M4 examination and USMLE Step 1 and Step 2 CK scores provides support for the utilization of the CDEM National EM M4 examination as an effective means of assessing medical knowledge for fourth-year medical students. Identification of students scoring lower on standardized exams allows for effective remedial efforts to be undertaken throughout the medical education process.

## INTRODUCTION

Assessment of medical students is complex yet essential to ensure that medical school graduates are prepared for residency training and future practice. Assessment is important to provide feedback to the learner in order to guide development and acquisition of milestones necessary for independent practice; to provide information to the educational program regarding effectiveness of the pedagogies; to provide a metric for stratifying the competency of applicants; and to protect the public by ensuring all graduates have attained the requisite level of competency required to progress to the next level of training.^1^,^2^ The Liaison Committee on Medical Education (LCME), the accrediting body for education leading to the MD degree, has established guidelines for the evaluation and assessment of medical students throughout the continuum of undergraduate medical education.^3^ These guidelines specify the use of formative and summative assessment methods to examine a variety of measures of knowledge, skills, behaviors, and attitudes (LCME, Functions and Structure of a Medical School).^3^ Clerkships typically employ a number of assessment tools including written examinations, oral examinations, direct observation, simulation, observed structured clinical examination (OSCE), oral presentations, and written reports. While the assessment of clinical performance can be influenced by examiner subjectivity, medical knowledge assessments are often more objective in nature and are an important outcome for curricular assessment and licensure.^4^

Despite the increase in the prevalence of required emergency medicine (EM) clerkships within medical schools a National Board of Medical Examiners (NBME) subject exam in EM did not become available until April 2013.^5^–^7^ In a 2007 survey of EM clerkship directors, 59% of respondents reported using an end-of-rotation final examination as a component of determining a final grade for the clerkship.^5^ A subcommittee of the Clerkship Directors in Emergency Medicine (CDEM) recently developed a high stakes, end-of-rotation examination that was released on August 1, 2011 to assess fourth-year (M4) medical students.^7^ This high-stakes examination was designed to assess the standardized objectives specified in the National EM M4 curriculum developed by CDEM to ensure a more consistent experience for students rotating in EM throughout the country.^8^ This national curriculum also led to the development of online self-study modules designed to offer core knowledge on the core topics in EM (http://www.cdemcurriculum.org/). The examination was developed by a national group of EM educators using published NBME item writing guidelines and was made available to EM clerkships at no cost using online testing software.^7^,^9^,^10^

Regarding medical knowledge, many core clerkships use the NBME subject (shelf) examinations as a component of the final clerkship grade to increase objectivity. For example, in a recent survey of Clerkship Directors in Internal Medicine 88% of clerkships required the NBME subject exam to be taken during the clerkship and 99% of those clerkships used the examination score in determining the final clerkship grade.^11^ The Alliance for Clinical Education recommends that “end of clerkship examinations should be part of the evaluation process for all medical students in core clerkships and should supplement clinical evaluations.”^12^ A written examination allows efficient assessment of both breadth and depth of medical knowledge without being impacted by the “halo effect” or tendency to overestimate clinical skills of students well known to them. Global rating scales of clinical performance have low inter-rater reliability and tend to reflect the faculty’s undifferentiated judgment of the student’s overall performance.^13^ A national standardized exam not only allows student-to-student comparison, but also assists clerkship directors in assessing curricular goals and determining whether rotation learning objectives were met. Standardized, multiple-choice exams are well designed to test medical knowledge, but do not necessarily address other Accreditation Council for Graduate Medical Education core competencies (e.g., communication skills, professionalism, complex medical decision-making) that are best assessed via more clinically-focused observational methods within a clinical environment.

Studies evaluating the correlation between student performance on NBME subject exams, USMLE exams and clerkship performance have produced variable results. Successful completion of USMLE Steps 1, 2, and 3 designed to assess knowledge, concepts, and skills essential for patient care is required for medical licensure leading to the practice of medicine without supervision.^14^ Moderate to large correlations have been demonstrated between performance on NBME subject exams and USMLE Step 1 and Step 2.^15^,^16^ Most studies correlating the relationship between USMLE performance and NBME subject exam performance analyzed the performance for a single clerkship at a single site. Family medicine and obstetrics and gynecology have demonstrated modest correlation of USMLE Step 1 and Step 2 scores with the NBME subject examinations.^17^, ^18^ In addition, Myles demonstrated that students failing Step 1 were more likely to fail the NBME shelf examination for obstetrics and gynecology and those performing in the bottom 25% of the NBME shelf exam were more likely to perform poorly on Step 2.^15^ Only one study was identified that studied the relationship between NBME subject examinations across multiple clerkships with USMLE Step 1 and Step 2 scores, which demonstrated moderate to large positive correlations (0.69 and 0.77).^16^ Ultimately, understanding the correlation between performance of students on a variety of measures of clerkship knowledge and skills and performance on the USMLE examination series will allow undergraduate medical educators to identify at-risk students and institute early inventions to ensure successful completion of licensure requirements.

The primary objective of this study was to describe the correlation of the National EM M4 end-of-rotation examination with USMLE Step 1 and Step 2 Clinical Knowledge (CK) examination scores at a single medical school.

## METHODS

After approval of the institutional review board, all students in the required M4 emergency medicine rotation at the Brody School of Medicine at East Carolina University from August 2011 to April 2013 were eligible to be included in the study (a total of 159 students). We compiled the examination scores of the National EM M4 exam for consecutive fourth-year medical students (classes of 2012 and 2013) taken at the end of a required emergency medicine clerkship. Files containing data regarding performance of visiting medical students (n=14) and files from students not providing consent from our own school (n=12) were excluded from the study, leaving 133 students in the study sample. We correlated the National EM M4 exam scores with first attempts of both USMLE Step 1 and Step 2 CK scores and the final clerkship grade. All analyses were completed using JMP Pro 10 Statistical Software (SAS, Inc. Cary, NC). Correlation coefficients and 95% confidence intervals are reported. We also used one way analysis of variance (ANOVA) procedures to examine relationships between final EM exam scores, final EM rotation grades and USMLE Step 1 and 2 CK exam scores.

The CDEM-developed National EM M4 exam was administered at the completion of a four-week required M4 emergency medicine clerkship and accounted for 25% of the final clerkship grade. In addition to the examination, the final clerkship grade was based on end-of-shift clinical evaluations (55%), patient and procedure log (10%), professionalism (5%), and an evidence-based medicine presentation (5%). The designation of Honors criteria was applied to students who scored at least 80% correct on the National EM M4 end-of-rotation exam, demonstrated superior performance on shift evaluations, participated in an EMS experience, submitted an evidence-based medicine paper, and performed procedures beyond the basic requirements. All students completed the EM rotation at Vidant Medical Center, a tertiary care center with the only Level I trauma center for the 29 counties it serves in eastern North Carolina. The emergency department (ED) has greater than 110,000 patient visits annually with students rotating in both the adult and pediatric EDs. M4 students maintain primary patient care responsibility with the assistance of either faculty or teaching resident assigned for direct supervision. Educational objectives defining both medical knowledge and procedural competencies are taught through simulation exercises (three hours weekly), procedural skill labs, CDEM curriculum online reading, and didactic sessions. These objectives, along with simulation cases and required readings, are based on the revised national curriculum recommendations for required M4 clerkships in emergency medicine as delineated by CDEM and published in Academic Emergency Medicine.^8^

Students at the Brody School of Medicine at East Carolina University are required to take the USMLE Step 1 after completion of their basic science courses during the M2 year and before beginning their third-year core clinical clerkships. USMLE Step 1 must be passed in order to complete the M3 year without delay. The USMLE Step 2 may be taken in the summer or fall of the M4 year. Successful completion of both the USMLE Step 2 Clinical Knowledge and Step 2 Clinical Skills is a requirement for graduation.

## RESULTS

Among 133 students, the mean USMLE Step 1 score was 226.8 SD 19.3. The mean USMLE Step 2 CK score was 238.5 SD 18.9. The mean score of the National EM M4 exam was 79.5 SD 8.0, compared to a national mean of 79.7 correct SD 3.89.^9^ The range of score for these 133 students was from 62 to 94, compared to the range for national administration of the exam of 40 to 98.^9^ With 59 students scoring below the median and 74 students scoring at or above, the median score was 80. For the 59 students who scored below the median on the EM national exam, the mean USMLE Step 1 score was 218.8 and the mean USMLE Step 2 CK score was 230.0. For the 74 students who scored at or above the median on the EM national exam, the mean USMLE Step 1 score was 233.4 and the mean USMLE Step 2 CK score was 245.2.

**Figure 1:**
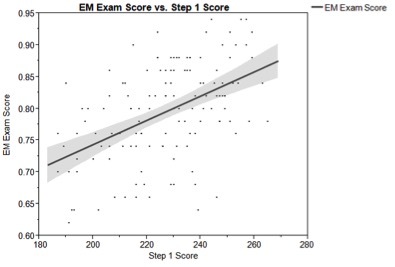
Correlation of National EM M4 Exam and USMLE Step 1 score. The bold line represents correlation and the shaded area represents the 95% confidence interval at each point. Correlation coefficient for the National EM M4 Exam and USMLE Step 1= 0.50. *EM*, emergency medicine; *USMLE*, United States medical licensing exam

**Figure 2:**
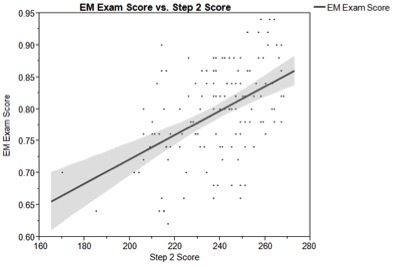
Correlation of National EM M4 Exam and USMLE Step 2 CK score. The bold line represents correlation and the shaded area represents the 95% confidence interval at each point. Correlation coefficient for the National EM M4 Exam and USMLE Step 2=0.47. *EM*, emergency medicine; *USMLE*, United States medical licensing exam; *CK,* clinical knowledge

National EM exam scores from these 133 students were correlated with USMLE examination scores using Pearson’s coefficient. Emergency medicine examination scores showed moderate correlation with both USMLE Step 1 (mean score= 226.8; correlation coefficient= 0.50; 95% CI [0.28–0.67], Figure 1) and USMLE Step 2 (mean score= 238.5; correlation coefficient= 0.47; 95% CI [0.25–0.65], Figure 2).

Final EM rotation grades were correlated with scores on the end-of-rotation EM examination as well as with USMLE Step 1 and Step 2 examination scores. The final EM rotation grade was weakly correlated with the EM exam score (correlation coefficient=0.19), the USMLE Step 1 exam score (correlation coefficient=0.08) and USMLE Step 2 CK exam score (correlation coefficient=0.04). None of these correlations were statistically significant.

**Figure 3:**
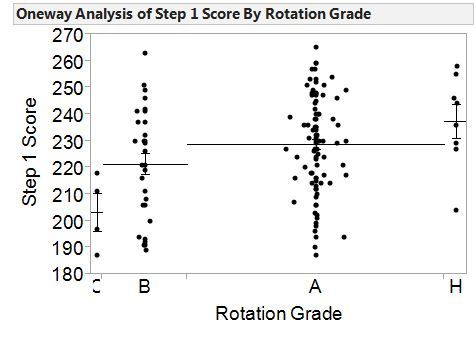
One way analysis of Step 1 Score by rotation grade. Individual data points are represented for each end-of-rotation grade category. Mean scores and standard errors are represented for each group by the horizontal lines. *H,* honors

**Figure 4:**
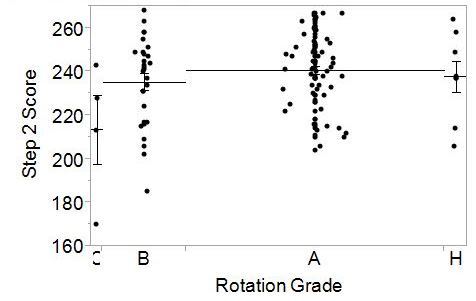
One way analysis of Step 2 CK Score by rotation grade. Individual data points are represented for each end-of-rotation grade category. Mean scores and standard errors are represented for each group by the horizontal lines. *CK*, clinical knowledge; *H,* honors

To better interpret the correlational analyses, we also used one way ANOVA to examine the relationships between final EM rotation grade, National EM M4 examination and USMLE Step 1 (Figure 3) and Step 2 CK scores (Figure 4). For students who achieved a final rotation grade of either A or Honors, the Step 1 scores were significantly higher than students whose final rotation grade was a C. For students who achieved a final rotation grade of A, their scores on USMLE Step 2 were significantly higher than students whose final rotation grade was a C. No other significant differences were found.

## DISCUSSION

Our results, the first published data comparing exam performance for this newly-created national examination for emergency medicine with national benchmarks, are encouraging in that medical students who performed well on USMLE Step 1 and Step 2 CK licensure exams during the first three years of the curriculum appeared to also perform well on the National EM M4 clerkship exam. These findings are also consistent with previous studies from other specialties that report correlations between NBME subject examinations and USMLE examinations.^15^,^16^

Demonstration of the association between performance on earlier and later knowledge examinations is particularly important as the National EM M4 examination is offered free of charge as compared to the NBME Emergency Medicine Advanced Clinical Exam. The NBME released an EM subject exam in April 2013, after the release of the National EM M4 exam in 2011.^19^,^20^ In an era of limited financial resources for medical education, decisions regarding test selection must consider not only the psychometric properties of the examination, but must take into consideration the financial implications for the institution.^21^ The psychometric properties of this nationally standardized exam need further study, but our results demonstrate that its ongoing use with M4 medical students completing EM rotations remains a viable option for assessing medical knowledge, particularly for schools who may struggle to find resources to use other similar national exams.

What are the implications of our findings for the educational process itself? Our results showed that students who scored lower on the national EM exam also scored lower on the USMLE licensing series. The use of scores on the USMLE series to predict performance on subsequent examinations is part of a larger conversation concerning predictive validity in general. Medical schools place a great deal of emphasis on the importance of high stakes, multiple-choice examinations. A plethora of studies concerning predictive validity exists, and it seems apparent that students who do well on multiple-choice exams early in medical school tend to be consistent in that performance across a variety of standardized exams.^22^,^23^ The same principle appears to be the case for student performance on USMLE Step exams, particularly Step 1. Our study illustrates the importance of using student scores from the continuum of USMLE Step 1, Step 2 and other local exams in the identification and strategic assistance for students who are “at risk” of poor performance on subsequent subject, in-service, and licensure examinations. We advocate the judicious use of student exam scores in this manner, in order to identify early in medical school those students who may have difficulty with exams given during core clinical clerkships such as our required fourth year EM rotation, and even standardized examinations given during residency training. A recent study reported that students performing poorly on one NBME subject exam were significantly more likely to fail USMLE Step 3 (OR 14.23) compared to peers without any subject exams 1SD below the mean.^25^ Many clerkship directors do not have knowledge of their students’ USMLE scores prior to the students’ arrival on a given rotation. While some faculty opposed to sharing those scores feel this information will provide bias against the student’s clinical performance evaluation, one must also consider how this information could be used more proactively by clerkship educators to provide enriched learning opportunities for students with a history of performance difficulties on standardized examinations.^25^–^27^ This strategy is particularly relevant for students from under-represented minority groups and/or students who have diagnosed learning difficulties.^28^–^30^ Developing a method for early identification and intervention for “at risk” students (however “at risk” is defined) may lead to improved performance on future licensure exams. This is critically important as many state licensing bodies are imposing limitations on the number of attempts required to pass USMLE or Comprehensive Osteopathic Medical Licensing Examination (COMLEX) examinations. Educational strategies such as administering a rotation pretest of medical knowledge may provide assistance to clerkship directors in identifying these “at risk” students in need of focused tutoring, mentoring, or other specialized assistance. Remedial strategies including mandatory lecture attendance or extended time with a preceptor have been shown to improve student performance on NBME clinical subject examinations.^31^,^32^ The goal of medical education should be to establish learning environments targeted at helping students achieve their maximum potential, including improved performance on medical knowledge exams. By considering the performance differences of students at different levels, EM educators can consider how the continuum of standardized exam results can be used proactively to meet the needs of students facing academic challenges. This latter need is especially critical for medical educators in Emergency Medicine, the majority of whom encounter medical students on clinical rotations during the latter part of the third year or the senior year. The critical need to remediate students prior to graduation may not be identified earlier, thus leaving this task up to EM faculty who teach students that have limited time remaining in their medical school experience.

## LIMITATIONS

Our study is limited by its enrollment of students at a single institution and the relatively small number of students included. From 2010–2015, an average of 8.3% of our students matched into EM or combined EM residency programs, consistent with the national average of 8.5% of students matching into EM and suggesting a representative sample of EM-bound versus non-EM bound students.^33^ While the majority of students take the USMLE step 1 at a similar period of time, students complete the required M4 EM rotation throughout their M4 year. The majority, but not all, of our students have also taken USMLE Step 2 prior to entering the M4 EM clerkship. This presents students with significant differences in both experience level and motivation. An additional potential source of error is the effect of rotation sequence and clerkship rotations selected during the M4 year. Previous studies have suggested that primary care rotations account for variance in USMLE scores and may represent significant content on USMLE Step 2, which is not emphasized on the National EM M4 exam. This may contribute to the slightly lower correlation between the national EM examination and USMLE Step 2. The correlation with USMLE Step 1 may more accurately reflect students’ overall test-taking ability and inherent knowledge (as opposed to specific knowledge obtained on the EM clinical rotation).

Larger studies are necessary to further examine the correlation of the National EM M4 examination with USMLE Step 1 and Step 2 scores, thus increasing our confidence in the construct validity of the National EM M4 exam. And, future research should also focus on gathering information about the clerkship learning activities provided to prepare students for the examination and the remediation strategies utilized for struggling students.

## CONCLUSION

Our study provides support for the validity of the CDEM National EM M4 Emergency Medicine examination as a means of assessing medical knowledge for fourth-year medical students on an EM clerkship. Future studies of examination performance should be designed to help identify students who can benefit from remedial efforts, and how those efforts can best be structured.
